# Supplementary material for: Female adolescents living with HIV telling their story through ‘my story’ book in Malawi: A visual methodology innovation
Source: PLoS One. 2021 Oct 15;16(10):e0257126. doi: 10.1371/journal.pone.0257126 (PMC8519441; doi:10.1371/journal.pone.0257126)
Supplement: S1 Dataset — (PDF) [file pone.0257126.s001.pdf]

CHAPTER NINE

STUDY CONCLUSION

9.1 Introduction

This final chapter summarises the study's key findings and interpretations to show its key contributions to knowledge in the field of HIV adolescent health with the use of 'my story' book. The implications of the findings for programme development, service provision and future research are described. This chapter is divided into three sections. The first section focuses on the key contributions to knowledge within the two overarching focus areas of the current study – the psychosocial and SRH experiences and challenges of growing up with HIV. The second section outlines the implications of the findings. The final section outlines the study's strengths and weaknesses.

The aim of this study was to determine the meaning of the experiences of growing up with perinatally-acquired HIV for young women aged 15-19 in Malawian context. The study aimed to explore the complexities of young women's lives from the perspectives of young women, their caregivers and service providers. The study further sought to examine young women's challenges in order to understand their health outcomes within the wider socio-cultural and structural context that shaped their experiences and options.

The study addressed the following research objectives:

To explore the psychosocial, sexual and reproductive experiences of growing up with perinatally-acquired HIV for the young women

- To determine the structural factors, perceptions, values and aspirations influencing the young women's experiences as they grow up with HIV to adulthood.

To explore the health, sex and relationship challenges for young women growing up with perinatally-acquired HIV and the impact of the current HIV related and SRH services

- To identify the psychosocial and SRH challenges of the young women as they are growing up to adulthood.
- To identify the issues and challenges encountered by caregivers as they look after the young women growing up with HIV.
- To identify the issues and challenges encountered by service providers as they provide health care to the young women growing up with HIV.
- To assess the impact of the existing health services on the needs of the young women as they grow up to adulthood.
- To identify the most important and realistic strategies in meeting needs of perinatally HIV infected young women as they grow up to adulthood in Malawi.

This study employed a case study approach. Individual in-depth interviews were the method of data collection. Forty-two initial semi-structured interviews were conducted with the three categories of the participants – young women, their caregivers and service providers. Three interviews for each 'case' comprising of a young woman (15-19 years), a nominated primary caregiver and a service provider making fourteen interviews per each category of participants. Ten follow up interviews were conducted with some of the participants regarding issues arising from initial interviews that required clarifications, but were not as structured or detailed as the initial interviews. A repeat interview was conducted with a caregiver (a sister to Ulemu), who became distressed as she was narrating about the nature of relationship with their aunt. 'My story' books which comprised of researcher-generated images depicting different life experiences and events and sentence completion exercise were used to facilitate interviews with the young women as they explained their experiences and needs basing on the chosen images. 'My story' book gave young women more control over the representation of their lived realities, thereby addressing unequal power relations between the researcher and the researched. The book allowed young women to communicate not only key events but also contexts, values and challenges as they explained the experiences in reference to the chosen images.

Since young women are considered persons with diminished autonomy, they are vulnerable to coercion or undue influence, thus the researcher ensured that their participation in the study was informative and non-coercive. Information sheets and consent forms were used in recruitment of the participants. Confidentiality, anonymity and respect for privacy were considered paramount as on-going issues at every stage from data collection to the write up of this thesis, especially in view of the stigma and secrecy surrounding HIV. Retroductive analysis was undertaken to penetrate to a deeper level of social reality by discovering the real mechanisms or structures that led to young women's experiences (Easton 2010). Within-cases and cross-cases analyses were undertaken to produce more contextually grounded, transferable findings. A focus on individual accounts within each 'case' was important to be able to view each case individually within its own context and remain true to the case study approach (Stake, 2006), as reflected in Chapter Four. Cross-case analysis was undertaken to examine how systematically themes and sub-themes (issues) cut across all the cases to identify similarities, differences, relationships and contradictions across the fourteen cases, then cross-case conclusions were drawn, as reflected in Chapters Five, Six and Seven.

9.2 Summary of key study contributions

This section summarises the key contributions to knowledge within the two main focus areas of the current study. The first area was concerned with the psychosocial experiences of young women and how they coped with their complex situations. The second area was on health, sex and relationship experiences and challenges, and how the existing health services impact on young women's experiences.

9.2.1 Psychosocial experiences and challenges in growing up with HIV

- Key contribution 1: Young women endure hidden pain and long suffering on their own, as a result of cultural silence.

The current study provides a new insight that most young women endured the hidden pain, long sufferings and the tragedy of the disease on their own, which was attributed to cultural silence. Cultural beliefs about discussing death and sexual issues (including contraception) with children in many Malawian cultures limited young women's chance of discussing and dealing with their intense feelings and challenges with the caregivers and service providers.

This was exemplified by young women's expression of unspoken grief as they explained their traumatic experiences of feeling neglected and abused for years.

- ❖ Key contribution 2: Loss of physical attractiveness generates negative attention from female peers, leading to marginalised and stigmatised experiences.

Almost all young women with visible marks of HIV infection that affected their physical appearance felt more marginalised among their female peers compared to their male counterparts, thus affecting their social identity. The study further adds that social rejection linked to changes in physical appearance was more prevalent from their female counterparts. This could be attributed to social values on ideal qualities and physical appearance for a woman, which collide with the reality of HIV infection. Hence, this presented risks for their sexual health as they sought love, acceptance and personal security in male relationships.

- ❖ Key contribution 3: Young women have agency to deal with their difficult situations.

The current study provides a new insight that these young women are active agents in their difficult situations as they build and use strategic relationships, particularly sexual relationships, as an economic strategy to boost their self-image and secure their livelihood. This signifies that young women have the agency and capacity to deal with their social challenges, limits and demands that make up their everyday lives.

- ❖ Key contribution 4: There are several factors within the family and health system that may promote resilience, agency and psychological well-being among the young women.

The current study identified that collaborative, positive, adolescent-centred, adolescent-service provider relationships, social capital (strong support networks with caregivers, peers or teachers – emotionally and financially), religious beliefs and access to SRH information and choices are key in promoting young women's resilience, agency and psychological well-being. These factors improved the young women's capacity to achieve their academic and vocational goals and resist peer pressure, thereby reducing their exposure to sexual risks. The literature is silent on the specific components that make up the significant patient-provider relationships, suggesting that more research is required in this area. The current study further adds that safe and supportive environment, youthful professionals, child counselling and

adolescent friendly skills are essential in understanding and embracing the social, moral and sexual issues dominating young women's lives hence effectively meeting their needs.

- Key contribution 5: Most caregivers need emotional and financial support themselves.

Living with the positive HIV status is challenging for both the young people and their caregivers (Punpanich et al. 2012). The current study provides insight that caregiving coupled with younger age, low socio-economic status, social rejection and personal insecurity overpowered the caregivers' will to care, productivity and economic security. Hence, it diminished the coping abilities for both the young women and their caregivers. Caregivers' inability to provide for the young women's needs increased young women's vulnerability. This clearly shows that most caregivers need support themselves.

9.2.2 Sexual and reproductive health experiences and challenges

- Key contribution 6: Young women engage in sexual relationships to seek love and acceptance, and to boost their self-image.

Prior studies show that perinatally HIV infected young people engage in sexual activities for pleasure and financial gains (Busza et al 2013, Hodgson et al. 2012, Li et al. 2010, Obare and Van der Kwaak 2010). The current study has extended these findings showing that young women also engaged in sexual relationships to seek love and acceptance and to boost their self-image. The HIV positive status and the visible marks of HIV infection elicited stigmatization by peers and the society, hence affecting young women's social status and identity. As such, they engaged in male relationships for companionship and/or as an economic strategy in attempt to boost their social status and self-worth as young women following the social rejections and loss of parental affection and support.

- Key contribution 7: The complexity of young women's lives, particularly around socio-economic realities and status, sexual relationships, gender power relations and normative social expectations, diminished their agency, decision making power and choices on sexual issues.

The current study has shown that multiple losses (including the loss of adult support) implied loss of belonging, limited or lost opportunities for young women to secure finances and advance academically. This diminished young women's self-image. Since this made them feel different from their female peers (in terms of socio-economic status and physical attractiveness), they attempted to regain that sense of belonging and sought love and acceptance through sexually attaching themselves to male partners. They also relied on men for material and financial support to boost their social status. However, they encountered gender power inequalities in these male relationships which limited their sexual agency (including bargaining power) and choices in sexual issues. This, coupled with cultural normative expectations, gives male partners a position of power concerning sexual issues and a correspondingly diminished ability among young women to make decisions about safer sex practices. As a result, despite young women having access to appropriate information, it became very challenging to adopt protective measures, to which they were already averse due to their longing for status through sexual relationships.

- ❖ Key contribution 8: Turning a blind eye to young women's sexual activities is a common response by both caregivers and service providers.

The current study has shown how caregivers and service providers turn a blind eye to young women's sexual activities. Both caregivers and service providers complied with their own cultural and religious beliefs, which hindered mutual communication on sexual issues with the young women. Service providers acknowledged that their reluctance was rooted in their lack of skills in youth-friendly health services and cultural and religious beliefs that reinforce abstinence. Most caregivers turned a blind eye to young women's sexual activities in exchange for financial or material gains for they were unable to provide for the young women's needs. In this context, most of them were unable to control young women's risky sexual behaviours, or it could also be a collective collusion to maintain face or a family's reputation.

- ❖ Key contribution 9: Ambivalences in priorities, values, preferences and perceptions affect young women's compliance to adult guidance and access to SRH services.

Prior studies found that status disclosure to a sexual partner can encourage adoption of protective measures (Obare and Van der Kwaak 2010, Birungi et al. 2009, Kadowa and

Nuwaha 2009). However, the current study provides a new insight that the attitudes, beliefs and actions of the others, dependence on the male partners for support and cultural and social normative expectations (gender inequalities) compelled young women to keep their status a secret. Despite caregivers and service providers emphasising the significance of abstinence, protective sex and HIV status disclosure particularly to sexual partners, the young women resisted. The study further adds that caregivers were more concerned with protecting their own families from bad reputation, social exclusion and legal repercussions if the male partners discovered on their own. In contrast, the young women feared social rejection and loss of their sources of support which assisted them to cope with their difficult situations and boost their self-worth. These ambivalences in views concerning sex, safer sex and status disclosure to partners signified differences in values and priorities between caregivers/service providers and the young women. These differences in turn affected young women's compliance to caregivers'/service providers' guidance and access to SRH education and services as they perceived other risks.

- ❖ Key contribution 10: HIV is mainly women's responsibility.

The current study has shown that HIV responsibility is mainly placed on young women. Prior study in Malawi (Reniers, 2008) revealed that women are likely to be divorced after their spouses learn of their HIV positive status, due to male dominance in sexual issues. The current study provides a new insight that apart from male dominance, the termination of the sexual relationships by male partners upon status disclosure was attributed to the fact that HIV responsibility was placed upon the female partners. Similarly, caregivers' concern and pressuring of young women to disclose their status to male partners than vice versa, uncovers lack of attribution of responsibility to men but rather placing the HIV responsibility on women.

- ❖ Key contribution 11: All HIV management centres under study still fall short of providing for young women's SRH needs

The current study provides an insight that the HIV management centres under study still fall short of providing what the young women need and an appropriate approach (the services that are appealing and friendly to the young women). Young women felt they were not being heard as individuals. Service providers fail to create an atmosphere in which young women can express their individual concerns and expectations or participate in decision making and

have their SRH choices respected. Although peer support groups promote psychological well-being (Johnston et al. 2012, Gately et al. 2007, Koch et al. 2004), the young women strongly felt that some of the universal SRH strategies employed in the centres, like group discussions and offering condoms to every adolescent are not solely appropriate in meeting their individual needs. In addition, those who become pregnant felt that they are discharged from teen club without being given an option for social support or are exposed to dominant and oppressive power relations with older women as they shared the antenatal services in different health facilities.

9.3 Implications of the study

The findings of the current study have several implications for the programme development, service provision and research.

9.3.1 Programme development and service provision

The findings from the current study can inform the practice of HIV care delivery and development of programmes relevant to young women growing up with HIV in diverse ways. These implications include interventions for: (i) the young women, (ii) their caregivers, (iii) the community, (iv) the service providers; and (v) the models of care.

9.3.1.1 Interventions for the young women

The findings revealed that young women are unable to communicate their intense feelings, hidden pains and experiences to caregivers and service providers due to cultural silence. Hence, their intense feelings and agony remained unaddressed within most families and the health systems. Lack of knowledge about young women's suffering left many caregivers even service providers struggling to deal with the practical psychosocial and sexual issues affecting the young women, leading to poor mental health and SRH outcomes. The counselling services available in the centres are reactive to situations like status disclosure, and/or deviant behaviours as reported by caregivers. These findings have two implications. First, the findings suggest that planned, proactive and on-going individual counselling sessions should be offered to young women to help them cope with loss, sexual rights, gender power roles/norms or a supplementary strategy like individual therapy to effectively help breach the deep-seated cultural silence. The service providers (including teen club coordinators) need to

further develop child counselling strategies and skills through continuing education or short training courses to effectively address young women's mental health needs. Young women's expectations of humane responses from service providers to their issues are equally significant. Second, HIV care services need to incorporate mental health assessment from routine screening to thorough assessment and provision of care.

The current study shows that young women engage in early sex for love, acceptance and support. It is evident that in spite of advising young women to disclose their status and refrain from sexual activity or use condoms, they still engaged in unprotected sex leading to unwanted pregnancies. As such, optimal SRH outcomes can be achieved only when young women's perspectives on ideal sexual health services are understood and considered paramount. Currently, service providers emphasise on abstinence, or young women are offered condoms without choice, yet most struggle with condom use with their partners, leading to unwanted pregnancies. The social construction of masculinity and femininity and the socio-economic characteristics shaped the factors that constituted barriers to protective behaviour to a large extent. These findings have three implications.

First, the current study suggests that SRH promotion efforts need to be in line with young women's own perceptions of their sexual health. Ignoring this perception is undermining the principles of empowerment that emphasise the significance of recognizing young women's own concerns (Laverack, 2005), and the SRH services will seemingly be irrelevant to young women. Therefore, service providers need to understand young women's priorities, values, preferences, expectations and goals in order to design risk reduction programs that are appropriate and mutually acceptable.

Second, the current study suggests that policy makers and NGOs need to be aware of young women's material needs, and get involved in empowering these young women with stable income generating activities or sources of income (not hand-outs). Thus, without empowerment on resource mobilization, guaranteed condom access alone is inadequate to reduce their risks for poor SRH outcomes. Economic opportunities through small scale businesses, academic and vocational achievements in the current study gave young women a position of power to desist being vulnerable and fostered their ability to exercise agency and become resilient. The need to empower the young women reinforces what the then

United Nations Secretary-General Kofi Annan emphasised in 2004 at an International Conference in Bangkok (Kofi, 2004), “what is needed is positive change that will give more power and confidence to women and girls. Change that will transform relations between women and men at all levels of society.”

Third, the current study suggests that the service providers (including teen club coordinators) need to further develop strategies and skills on resource mobilization and provision of contraceptives to young women through continuing education or short training courses. This in turn enables them to conduct regular trainings with young women to effectively access contraceptives other than condoms and empower them with ideal resource mobilisation strategies.

9.3.1.2 Interventions for the caregivers

The current study shows that the majority of the caregivers encounter various emotional, physical, social and financial difficulties that in turn influence the care and support they give to young women. In addition, caregivers with low socio-economic status also struggled to control young women’s sexual behaviours. Therefore, these findings suggest that caregiving must be acknowledged as a central part of the response to the epidemic. Caregivers require a range of financial, psychosocial, medical and social support themselves as most of them are young, over-stretched with the caring responsibilities, and unable to provide for the young women’s needs. There is need to provide programmes that will build up caregivers’ local social supportive networks, and training that will enable caregivers to identify ideal resource mobilisation strategies. This can create economic opportunities for caregivers particularly female caregivers who are themselves struggling with the existing gender power relations.

The current study demonstrates that most biological caregivers, particularly the females, are marginalised and blamed for passing on the virus to their young people or for breaching morality. This finding suggests that the nature and impact of this stigma needs to be acknowledged and addressed in healthcare practice and policy, particularly its ability to influence parental capabilities to support the young women and seek support from the health facilities or communities where necessary. The caregivers need to be assisted with how they can deal with HIV in an adolescent–friendly, age-appropriate way through training or regular joint meetings with the services providers focusing on:

- ❖ The complex practical issues affecting young women. These include the normative expectations, different priorities, values and develop realistic strategies regarding risk reduction and economic security among the young women. Instead of distancing themselves from young women's social realities, greater attention to young women's interests, priorities and values (social and medical) is thus crucial to promote their compliance to adult guidance.
- ❖ Communication skills around status disclosure and sexual issues with their young women without focusing on blame or shame, and to identify practical issues affecting the young women and refer them appropriately.
- ❖ Caregivers' own emotions and fears regarding the young women and balancing the benefits and difficulties of parental disclosure and support or parent-child discussions on challenges within the specific personal, family and cultural contexts.
- ❖ Understanding more about factors that influence status disclosure within the family context which may allow for development of family-centred interventions like establishment of family clinics that improve coping capacity within the family unit and beyond.

9.3.1.3 Intervention for service providers

The current study has shown that despite some young women being sexually active and others becoming pregnant, some service providers were still reluctant to discuss sexual issues with the young women or to offer them SRH services, particularly injectable contraceptives. The findings show that cultural normative expectations locate service providers at a critical intersection between cultural and social values which promote sexual abstinence until marriage, and the reality of young women's premarital sex and desire to use contraceptives. These findings have two implications.

First, the study suggests that service providers need to acknowledge that they are key in embracing the young women's complex life, and empowering them to acquire decision making and negotiation power and skills on sexual issues through SRH education and services. Therefore, instead of controlling young women's sexual behaviours and access to the services, service providers need to network and establish collaboration with caregivers. Both service providers and caregivers must realise that young women's health related choices are different

from their own and should not be dismissed as wrong or irrational, but rather they should be effectively acknowledged and addressed. Both need to empower young women with the necessary information to enable them to balance rights and responsibilities, exercise agency, make informed decisions about their sexual health and contribute to their quality of SRH well-being.

Second, the study suggests that it is critical that cultural, social and religious norms attached to young women's sexuality and access to SRH services be incorporated in all undergraduate trainings for health professionals and continuing education should be offered to assist all service providers to deal with the realities of adolescent health and the changing picture of adolescent sexuality even in the context of chronic illness. Service providers should be trained or reoriented to undertake their work without becoming 'parents' to young women in order to promote open discussions on sexual issues. What is needed by the young women goes beyond simple provision of SRH services, but includes assisting them acquire life skills such as capability to communicate, negotiate safer sex and the right to make sound decisions.

9.3.1.4 Implications on models of care

Basing on the National SRH and Rights Policy, WHO's recommendations for adolescent friendly services (WHO, (2012) and the findings of the current study, it is clear that SRH services in all the centres under study still fall short of providing what the young women need (i.e. appropriate care), particularly with regard to services that are appealing and friendly to young women. In this context, the current study suggests that all the health services should be made adolescent-friendly; adolescents should access the services they need to make a positive contribution to their health. The SRH services should be accessed by all young women from the age of 15 years, as recommended by the National SRH and Rights Policy and be provided in the right manner with respect, without getting the young women into trouble with their parents or caregivers. Young women's values and preferences be respected and confidentiality about sexual behaviour should be protected.

In the current study, several young women were sexually active and five out of fourteen had experienced a pregnancy. The current study investigated their pregnancy outcomes and one young woman has had two children who acquired HIV perinatally (the second child is dead).

The findings show that pregnant young women disappear from teen club meeting for fear of being stigmatised by their peers and some service providers. They access antenatal services in other health facilities and reappear at HIV management centres or at teen club after delivery of their babies. Effective prevention of mother to child transmission (PMTCT) is a critical component of antenatal care (ANC) for the pregnant young women to reduce vertical transmission. This study suggests that the current practice of referring young women to other facilities for antenatal services be reconsidered. The ANC should be integrated in HIV management strategy to reduce social reproach as currently young women are being subjected to dominant and oppressive power relations with older antenatal women when they share the services. Service providers should be able to identify pregnant young women early and ensure that they access a full range of antenatal and PMTCT services. The findings also recommend that the National HIV and AIDS Policy and SRH and Rights Policy need to consider young women who acquire HIV perinatally as one of the priority areas in provision of impact mitigation services.

It is evident in the current study that young women encounter several challenges as they access SRH education and services in the centres under study. As such, they pointed out significant and practical SRH issues for the improvement of their SRH outcomes. The seven central issues regarding SRH education and services were identified and these include the need to:

- be heard as individuals, not as a group (like during teen club meetings).
- have their complex practical issues given a priority beyond the medical scope.
- have their caregivers supported with young women's practical needs, including adolescent sexual health and ideal resource mobilization strategies, rather than just focusing on control of their sexual issues.
- be provided with an opportunity to make their own decisions regarding their sexual well-being in supportive, trustworthy relationships, and safe and friendly environments, to access services they need in light of their diverse challenges.
- be reviewed by service providers with adolescent-friendly skills, who can understand their sexual trajectory without judgemental attitudes, thus enabling them open up on their sexual issues and freely access the SRH services.

- ❖ be in contact with specific service providers - suggestive of continuity in care as it can facilitate problem-solving. The young women felt that continuity in care could significantly enable service providers to follow them up on personal and sexual issues, unlike in the current situation, whereby they have to be seen by different service providers, creating disruptions in their care and follow-up.
- ❖ have a separate support group (teen club) for teen mothers to share experiences and challenges of parenthood, and individually or collectively to mobilise resources to reduce their vulnerability.

9.4 Study strengths and weaknesses

9.4.1 Study weaknesses

Young women and caregivers approached and recruited in the current study were those who were attending multidisciplinary centres where HIV related health services were readily available, and the majority of service providers had specialised training and experience in the field of HIV and AIDS. Young women who were lost to follow-up and accessing services in peripheral facilities who are likely the most marginalised and most vulnerable to social exclusion and inequalities were not approached because were harder to reach. These were likely to have different experiences and challenges of growing up with perinatal HIV. Nevertheless inclusion of accounts of young women from impoverished families or who lived in rural settings and encountered difficulties visiting the facilities due to financial constraints provided insight into these complexities of accessing the services. The use of a number of research methods also assisted the researcher to ably consider the congruence and complementarity of each participant group and researcher's field notes with the data from the participants' in-depth interviews provided a comprehensive overview, but more in-depth study of particular groups is needed (Greene and McClintock, 1985).

The study includes only experiences of young women. Although they cited numerous instances of male beliefs and behaviours, the findings may not adequately represent the male perspectives. It must be noted that the experiences of the young men could be different or could share some similarities. Further studies of young men and the comparison between the males' and females' experiences are recommended for future researchers.

9.4.2 Study strengths

The case study approach and use of 'my story' book enabled discovery of complex processes and structural realities of the young women's lives at individual, family and health facility levels that may not have been attained through other approaches. The approach enabled the researcher to focus holistically on young women as it encompassed all the key individuals involved in provision of their care within the family and the health systems. Hence, multiple perspectives yielded in-depth understanding of young women's psychosocial and sexual experiences of growing up with perinatal HIV.

While acknowledging that in using 'conventional' data collection techniques, young people were sometimes reluctant to discuss their sexuality and avoided direct questions on sexual issues (Busza et al., 2013), the innovative 'my story book' approach designed for the current study encouraged young women's openness on sexual issues. The approach addressed unequal power relations between the researcher and the researched, hence yielding powerful and rich data on young women's sexual experiences. Young women were able to communicate not only key events but also contexts, values and challenges including sexual issues as they explained the experiences in reference to the chosen images.

9.5 Conclusion

This study is unique because it is the first study to determine the psychosocial and sexual experiences and challenges of the young women with perinatally-acquired HIV holistically using 'my story' book (within the family and the health systems), based on the underlying structures and mechanisms in real-life contexts. Use of 'my story' book has demonstrated a contextual understanding of interpersonal dynamics, mechanisms and structural realities influencing the sexual agency as a cornerstone of adolescent sexual health (particularly among young women growing up with HIV). Disseminating these findings in the HIV management centres, through publications and forums like national and international conferences will inform the policies and health practices on appropriate and realistic strategies of supporting the young women within the family unit and health systems in order to promote optimal mental health and SRH outcomes.
