# Supplementary material for: Female adolescents living with HIV telling their story through ‘my story’ book in Malawi: A visual methodology innovation
Source: PLoS One. 2021 Oct 15;16(10):e0257126. doi: 10.1371/journal.pone.0257126 (PMC8519441; doi:10.1371/journal.pone.0257126)
Supplement: Attachment — Submitted filename: Comments for Manuscripts.docx [file pone.0257126.s002.docx]

Comments

This is a timely manuscript that addresses innovative, youth-friendly approaches with adolescents and openly discusses their complexities and sexuality. I have made the following observations and comments:

1. There are repetitions in manuscript which can be catered for if there is a stand-alone paragraph on 'what' and 'how' the innovative visual qualitative approach, the 'my story' book has been used at global, regional levels and particularly Malawi. This will fit best as part of the background and in the objectives
   a. The paragraph or two may be put in the background, before the methods section
2. Please confirm the ages of the participants. The abstract says 16-19 years, while the methods section refers to 15-19 years.
3. The authors should confirm demographic information for caregivers and service providers.
   a. It is also not clear how these were sampled and recruited.
4. The authors might need to consider to move the paragraph on the rationale of the study to the background section to situate the gap, objectives etc.
5. There is a need for a stand-alone paragraph about authors talking about their positionality. There is a need to acknowledge the power-related issues that come with the role of older woman/nurse/midwife working with adolescents living with HIV.
6. Please specify what 'other means of survival' entails.
7. There is a need for the authors to dedicate a paragraph on recruitment of adolescents, their caregivers, health workers and the advisory committee, 2) data collection processes using 'my story' book should be laid out, i.e. using images, stickers, and completion of 'my storybook'; including the use of in-depth interviews, the key examples of questions presented to adolescents/
   a. The authors may consider presenting their research questions rather than figure 1 and 2 as they tend to read more like results.
8. Please provide the names of districts, 'multi-disciplinary centres', health facilities providing HIV care
9. Please specify the free websites or public domains that were accessed and their URL for the images presented in the manuscript
   a. Specify if the authors' used researcher generated images or internet-based.?
10. Include the paragraph on the advisory committee under data analysis
11. There is a need for the authors to reduce the repetitions on the importance of using the 'my story' book approach'. It will help to make it succinct and coherent
12. There are a few grammatical errors in the results section, discussion sections
