# Supplementary material for: Female adolescents living with HIV telling their story through ‘my story’ book in Malawi: A visual methodology innovation
Source: PLoS One. 2021 Oct 15;16(10):e0257126. doi: 10.1371/journal.pone.0257126 (PMC8519441; doi:10.1371/journal.pone.0257126)
Supplement: Attachment — Submitted filename: RESPONSES TO REVIEWER 2 COMMENTS.docx [file pone.0257126.s003.docx]

**RESPONSES ON COMMENTS AND OBSERVATIONS MADE ON THE MANUSCRIPT**

**BY REVIEWER 2**

| **SERIAL NO** | **COMMENTS/OBSERVATIONS** | **RESPONSE** | **PAGE NUMBER** |
| --- | --- | --- | --- |
| 1 | - There are repetitions in manuscript which can be catered for if there is a stand-alone paragraph on 'what' and 'how' the innovative visual qualitative approach, the 'my story' book has been used at global, regional levels and particularly Malawi. - This will fit best as part of the background and in the objectives - The paragraph or two may be put in the background, before the methods section. | Repetitions have been reworked/removed  The paragraph has been merged with the background information.  Done | 1-3 |
| 2 | - Please confirm the ages of the participants. The abstract says 16-19 years, while the methods section refers to 15-19 years. | Age range has been corrected to 15-19 | Abstract |
| 3 | - The authors should confirm demographic information for caregivers and service providers. - It is also not clear how these were sampled and recruited. | Sampling and recruitment process of caregivers and service providers included | 5 |
| 4 | - The authors might need to consider to move the paragraph on the rationale of the study to the background section to situate the gap, objectives etc. | Done | 1 |
| 5 | - There is a need for a stand-alone paragraph about authors talking about their positionality. There is a need to acknowledge the power-related issues that come with the role of older woman/nurse/midwife working with adolescents living with HIV. | Included | 6 |
| 6 | - Please specify what 'other means of survival' entails. | The paragraph deleted as the section focused on methodology approach |  |
| 7 | - There is a need for the authors to dedicate a paragraph on recruitment of adolescents, their caregivers, health workers and the advisory committee, - 2) data collection processes using 'my story' book should be laid out, i.e. using images, stickers, and completion of 'my storybook'; including the use of in-depth interviews, the key examples of questions presented to adolescents/ - The authors may consider presenting their research questions rather than figures 1 and 2 as they tend to read more like results. | Done  To avoid unnecessary repetitions aspect of advisory committee still remains on page 11  Done  Corrected and figures 1 and 2 removed | 4-6  11  7  7 |
| 8 | - Please provide the names of districts, 'multi-disciplinary centres', health facilities providing HIV care. | Done | 4 |
| 9 | - Please specify the free websites or public domains that were accessed and their URL for the images presented in the manuscript. - Specify if the authors' used researcher generated images or internet-based.? | The indicative images were extracted from the copyright free websites such as Wikimedia Commons. The definitive images were researcher generated images captured after pretesting indicative images with female adolescents living with perinatally acquired HIV at a different health facility offering HIV services, to determine their applicability and relevance in the Malawian context. (the captured images – young women, service providers gave written consents for the images) | 3 |
| 10 | - Include the paragraph on the advisory committee under data analysis | Done | 11 |
| 11 | - There is a need for the authors to reduce the repetitions on the importance of using the 'my story' book approach'. It will help to make it succinct and coherent. | Repetitions removed |  |
| 12 | - There are a few grammatical errors in the results section, discussion sections | This has been worked and proof reading done throughout the document. |  |
