# Supplementary material for: Female adolescents living with HIV telling their story through ‘my story’ book in Malawi: A visual methodology innovation
Source: PLoS One. 2021 Oct 15;16(10):e0257126. doi: 10.1371/journal.pone.0257126 (PMC8519441; doi:10.1371/journal.pone.0257126)
Supplement: Attachment — Submitted filename: RESPONSES TO ACADEMIC EDITORS SUGGESTIONS MADE ON 261120.docx [file pone.0257126.s004.docx]

**RESPONSES ON ISSUES RAISED ON THE MANUSCRIPT**

**BY THE ACADEMIC EDITOR**

| **SECTION** | **COMMENTS/OBSERVATIONS** | **RESPONSE** | **PAGE NUMBER** |
| --- | --- | --- | --- |
| Title page | - For the listing of authors remove the period after Malawi (in two places) - For the correspondence section state: “… is a University Lecturer, Dean of Faculty of Nursing Studies at Kamuzu College of Nursing, University of Malawi, a consultant, and an expert in Adolescent Health, Sexual and Reproductive Health (SRH), Gender Issues, Human Immunodeficiency Virus (HIV), and Acquired Immune Deficiency Syndrome (AIDS) and conducts research with young people based in Malawi. | The period after Malawi is removed  This is corrected | Title page |
| Abstract | - Replace the word ‘Background’ with ‘Introduction’. - Last sentence of introduction: remove words ‘gain voice’. - First sentence of methods: replace ‘living with HIV share’ with ‘living with HIV to share’. - Second sentence of methods: replace ‘researcher appreciate’ with ‘researcher to appreciate’. - Last sentence of conclusion: replace ‘one-to-one’ with ‘one-on-one’. | All suggestions have been worked on. | 2 |
| Introduction | - Instead of using ‘Background’ refer to this section as the ‘Introduction’. - First paragraph: spell out ‘SRH’ the first time that you use it. - Third sentence of first paragraph: Break down into 2 sentences since this is a long sentence. - Middle of first paragraph: replace ‘grow to adulthood’ with ‘transition to adulthood’. - End of first paragraph: replace ‘articulate the realities of lives including’ with ‘in articulating the realities of their lives, including’. - Second sentence of second paragraph: replace ‘that becomes difficult to be completely bridged.’ With ‘and it can become difficult to bridge this gap.’ - Middle of second paragraph: replace ‘mature female researcher’ with ‘adult female researcher’. - Middle of second paragraph: replace ‘perceive that what they’ with ‘ensure that what they’. - Middle of second paragraph: replace ‘influence policy’ with ‘as well as influence policy’. - Middle of third paragraph: replaces ‘However, my story’ book’ with ‘However, ‘my story’ book’. - First sentence of fourth paragraph: replace ‘formed interview’ with ‘formed the interview’. - Final paragraph of Introduction: replace ‘ensuring the researcher’ with ‘ensuring that the researcher’ - Very end of final paragraph of the introduction: revised to ensure that reader is absolutely clear that this paper is about the ‘my story’ book methodology and not about the content of the interviews. You can do this by starting the last sentence of the introduction to say: ‘The goal of this paper is to…’ | The whole section has been reworked and all suggestions made have been addressed  Done in a sentence starting with, …..This pattern | 3 - 5  3 |
| Methods | - Change title of this section to ‘Materials and Methods’. - Remove any mention of the names of clinical sites to protect confidentiality. You should refer to the size of the patient population, scope of services (e.g. only pediatrics? Only HIV?). Refer to urban versus rural sites (e.g. 2 urban and 1 rural site). - Under ‘recruitment and sampling’: replace ‘imposed to reduce any perceived additional’ to ‘implemented to reduce any additional’. - Under ‘recruitment and sampling’: replace ‘Out of the 20 adolescents, who’ with ‘Out of the 20 adolescents who’. - Under ‘recruitment and sampling’ specify out of the 14 the number who are from rural versus urban areas. - For consent indicate that everyone 18 and above provided written informed consent (no need to include verbal). For those under 18 indicate that the parent or legal guardian provided consent and the adolescent study participant provided assent (in this order). - Under ‘recruitment and sampling’: remove “(as outlined in PLOS consent form)”. - Under ‘recruitment and sampling’ (last paragraph of this section: replace ‘they operate in’ with ‘they operate’. - Last paragraph, recruitment and sampling: rephrase where it states ‘mature lady’ and include term adult. This last section should be rephrased as follows: ‘Based on her own experience as an adult female and mother, the researcher felt that older adolescents had a wider experience than younger adolescents, consistent with existing literature [provide at least 3 references]. Therefore, the researcher decided to include older participants, particularly ages 15-19 based on the age range she felt the young women could have broader experiences and be open to discuss issues like sexual behaviours.’ - Under ‘data collection using ‘my story’ book’: replace ‘formed ‘sentence’ with ‘formed the ‘sentence’. - Under ‘data collection using ‘my story’ book’: It is unclear to the reader what sections of the story book are included as part of an interview with the researcher and what part they fill in by themselves (if that occurs at all)? - Under ‘data collection using ‘my story’ book’: Where it states the majority of adolescents filled all sections of the story book, indicate the actual number. - Under ‘data collection using ‘my story’ book’: Is there any data that is not interview data (e.g. the participant just ‘filled it out’?). If yes, please indicate how this data was included, e.g. was it typed into files to be included in the qualitative analysis? - Under ‘data collection using ‘my story’ book’, last paragraph: replace ‘interviewing process’ with ‘interview process’. - Data analysis section: should be limited to 3-4 paragraphs maximum (do not include bullet points in this section). - Data analysis, first paragraph: replace ‘researchers view’ with ‘researchers to view’. - Data analysis: remove Table 1 and just state that the results related to the content are published elsewhere (and state reference) or that they are forthcoming (if not yet published). - For methods: make it clear to reader how data were collected for caregivers and health care providers. For example, if there are in-depth interview guides state that and there is audio-recording, etc., provide this detail, and so forth. | Changed  All names have been removed  All suggestions have been made……  done  The number of those from the rural (4) versus urban (10) has been specified  This suggestion has been worked on  Removed  Done  Rephrased  Done  This information has been incorporated under data collection – second sentence  Actual number of 13 is indicated  No…all data including the filled sentence completion exercise/responses which were linked to the chosen images., adolescents were requested to elaborate during interviews.  Done  Data analysis section has been shortened to 4 paragraphs and all bullets have been removed.  Replaced  Table I is removed and a statement……results related to content are forthcoming has been included……  Details of data collected from caregivers and service providers are provided in paragraph 3 under data collection. | 5  6  7  7  6  8  8  9  9  9  10  10-12  11  11  10 |
| Results | - First sentence of first paragraph: replace ‘Through sentence’ with ‘Through the sentence’. - Under ‘creating meanings’: it is not clear if the loss of parental affection is related to death of a parent?  Try to make this more clear. - Under ‘creating meanings’: replace ‘were viewed as competent’ with ‘were competent’. - Under ‘creating meanings’: for the quote that says ‘I got connected to him’ remove the extra space. - Under ‘creating meanings’: for paragraph starting with the word ‘Similarly’, remove the space before this word. - Under ‘creating meanings’: replace ‘In contrary, majority’ with ‘In contrast, the majority’. | Done  Due to death of a parent(s) added  Replaced  Space removed  Space removed  Done | 12  13  13  13  13  14 |
| Discussion | - Paragraph before limitations: replace ‘images, how did the images impacted on the range and depth of data collected overall, helped’ with ‘images, and how the images impacted the range and depth of data collected overall, which helped’. | The paragraph has been reworked and impact of images on range and depth of data collected included | 18 |
| Limitations | - Remove this title and just start this paragraph with: ‘There are a number of limitations in the current study’. - Remove ‘(taking into account their shorter attention spans)’. - Refer to possible implications of not including participants who were too sick or unwell (e.g. implications for empowerment?) - Refer to possible implications of only including adolescents in which a caregiver and health care provider could also be recruited. | Done  Done  Possible implications of not including unwell/too sick participants and those whose caregivers and service providers refused to participate have been included. | 19  19 |
| Overall | - References in the text are not in Vancouver format. Instead of XX et al., 2019, you should indicate the number in brackets, e.g. [5]. - References at end should all be consistent and in Vancouver format and in one size font. [Note: HTML markup is below. Please do not edit.] | Done | 22-23 |
|  | While revising your submission, please upload your figure files to the Preflight Analysis and Conversion Engine (PACE) digital diagnostic tool, <https://pacev2.apexcovantage.com/>. PACE helps ensure that figures meet PLOS requirements. To use PACE, you must first register as a user. Registration is free. Then, login and navigate to the UPLOAD tab, where you will find detailed instructions on how to use the tool. If you encounter any issues or have any questions when using PACE, please email PLOS at [figures@plos.org](mailto:figures@plos.org). Please note that Supporting Information files do not need this step. | Images uploaded to PACE |  |
|  |  |  |  |
