# Supplementary material for: Female adolescents living with HIV telling their story through ‘my story’ book in Malawi: A visual methodology innovation
Source: PLoS One. 2021 Oct 15;16(10):e0257126. doi: 10.1371/journal.pone.0257126 (PMC8519441; doi:10.1371/journal.pone.0257126)
Supplement: Attachment — Submitted filename: MWalabu 2.docx [file pone.0257126.s005.docx]

The authors have made a lot of helpful revisions and much improvement is seen in the manuscript as a result. There remain, however, some issues with the methods and the results sections that need rectifying. Lastly, the length of the manuscript can be reduced by thorough editing, which can also enhance the readability and cohesion in some areas.

1. Ethics statement
   a. I can see that assents for adolescents under 18 were obtained. I am wondering if parental informed consent (not permission) was not obtained for the younger ones.
2. Introduction
   a. Please include the year in the second sentence.
   b. Please specify the type of support for adolescents in HIV programs. This should also link to the succeeding sentence for cohesion i.e. who benefits from these services as far as age is concerned (since you are talking about transition)? Is there any evidence that highlight the current status of issues surrounding transitioning into adult care? Consider the articles from Malawi that discuss health services eg content in number 2 and 4 of your bibliography and this article- “Uptake of health services by youth living with HIV: a focused ethnography”.
   c. Consider re-organising sentences in the first paragraph to support the main topic, which I suppose is the epidemiology of HIV and SRH. For instance, the sentence “Yet, young people are frequently reported to be reluctant to discuss sexual matters….” does not seem to link well with the rest in this paragraph. Likewise, the sentence on lack of knowledge on experience is not supporting the SRH needs as stated in the former sentence. Ultimately, the content does not strongly highlight the need for an innovative data collection tool. I recommend a separate paragraph that can discuss challenges encountered while accessing/providing SRH including communication hierarchies and barriers to strengthen the problem statement (this was also highlighted in the first review). This should also provide a better transition into the next paragraph, which is discussing communication techniques.
3. Considering there are 4 authors, pay attention to how you are using ‘the researcher’ throughout the document- maybe lead author?
4. Consider moving paragraph 3, 4, 5, 6; which are more of procedures than an introduction, into the methods section.
5. How many experts were consulted?

**Methods**

6. Consider looking into the information provided under on Ethics statement and Ethics approval and consent to participate.
   a. Was parental consent sought for adolescents below 18 years of age? Please clarify.
7. Under recruitment and sampling:
   a. Consider substituting the second sentence with a statement on how and where the service providers were recruited from.
   b. On inclusion criteria: did literacy matter in this study?
   c. Consider merging the first part of paragraph 1 with paragraph 2 (discussing recruitment), and inclusion criteria should come in a separate paragraph,- for cohesion
   d. Consider moving findings in paragraph 3 and 4 into the results section e.g. Six reported living with their biological parent(s) (father/mother), three were married e.t.c.
   e. I feel working as a nurse/midwife or affiliation with an HIV management centre does not affect the relationship with both genders- please specify the meaning here. This paragraph can also be summarised and shortened.
   f. In general, there is a lot of information that is not suitable for this section. Consider relocating such information. This section is also long, it can be reduced to 2 paragraphs at most.
   g. The authors should consider highlighting reasons why health workers and parents were included in a study that was examining data collection methods on youth- you have talked about triangulation later in the document, maybe this should come earlier?
8. Data collection
   a. Please clarify which transcript was audited (the original or the translated)?
   b. How was translation bias managed considering only one person was involved in the process?
   c. This fact is repeated ‘The researcher transcribed all the recorded interviews verbatim soon after each interview.”
9. Data analysis
   a. The authors have mentioned that codes, subthemes and themes were identified but the procedures undertaken to analyze data are not clearly coming out for reproducibility. Could the authors make this section clearer? *(This was also recommended in the previous review).*
   b. How was the data handled considering it came from different sources? Where/when did the merging/triangulation of findings occur? *(This was also highlighted in the previous review)*
10. Results
    a. The second paragraph is not clear, consider rephrasing.
    b. Please maintain consistency with the wording in the themes
    c. The theme confirmatory and complementary is not quite relatable to the content, which is mostly on disclosure of HIV status and associated prejudice. It this the confirmation of adolescents' perspectives of disclosure of HIV with their caregiver’s views? If this is the case, I recommend renaming the theme since all other themes have confirmatory information from multiple sources.
    d. ‘Among our key research questions was the issue of status disclosure particularly’ contradicts the aim of the study. Did you mean interview questions? *(this was also queried in the last review)*
    e. Consider moving the last sentence of theme two to the discussion section
    f. Is it ‘power relations in inter-generational research’ or ‘enabling intergenerational research?’
    g. Similarly, the first two sentences under the last theme are more suitable in the discussion section information of disclosure from caregiver
    h. Again, the title for theme three (research) does not relate to the content (perceived disagreement). Consider renaming the theme. Please add quotes from caregivers and health workers to support the claim of inter-generational dissonance.
11. Discussion
    a. Please cite the first sentence
    b. Please avoid repeating phrases or sentences e.g. it can be methodologically difficult for an adult researcher to find techniques to listen to the young people’s voices in research
    c. The discussion section is much improved and reads well.
