# Supplementary material for: Female adolescents living with HIV telling their story through ‘my story’ book in Malawi: A visual methodology innovation
Source: PLoS One. 2021 Oct 15;16(10):e0257126. doi: 10.1371/journal.pone.0257126 (PMC8519441; doi:10.1371/journal.pone.0257126)
Supplement: Attachment — Submitted filename: Review comments 2.docx [file pone.0257126.s006.docx]

FEMALE ADOLESCENTS LIVING WITH HIV TELLING THEIR STORY THROUGH ‘MY STORY’ BOOK IN MALAWI: A VISUAL METHODOLOGY INNOVATION:

I would like to commend the authors for thoroughly revising the paper. However, the paper cannot be accepted for publication in the current form. There are minor changes to be considered and a few areas that need to be strengthened for coherence.

1. There are a few paragraphs where the authors repeat their ideas.
   a. Pages 9 and 19 when the authors refer to ‘safe places’.
   b. Pages 9 and 10 as they talk about ‘keeping the thematic log.’
2. The paragraphs in the introduction need to be strengthened.

I appreciate the arguments from Kellett, Chambers and the appropriateness of using the ‘my story book’.

a. There is a need to know more about the concept of ‘my story book’ or the ideas of ‘memory book’.
b. How did the ‘book’ work in the past? How they have been used global, sub-Saharan Africa and Malawi are still missing.
c. ‘How?; When? and where? These concepts have been used, will strengthen the gap that Teachman and colleagues and Wills and colleagues presented.
d. As it is, the paragraphs from pages 4-7.
   i. The concept of my story books…
   ii. The main themes of my story books…
   iii. The discussion held with the researcher…

The above paragraphs leave the reader hanging and need real-life supporting evidence from the region or elsewhere. In addition, they continue to read like a paragraph that is part of the methodology section rather than the introduction. We need a solid and convincing introduction section.

3. The methodology is still too long and it shares some results too.
   a. On page 7, the authors refer to 14 adolescents who completed the story book.
   b. The researchers/author’s positionality should be reflected as part of data collection rather than recruitment and sampling.
4. The results section.
   a. The first paragraph can be dedicated to demographic information and the emerging themes or contribution to knowledge.
   b. Rather than reflecting on what the paper highlights, please rephrase and talk about key themes.
   c. The case of Tawina may follow Ziliwe or Zaiwa’s narratives to maintain coherence.
5. Discussion section
   a. Please include tangible evidence from the region (sub-Saharan Africa) on how using memory books/my story book or ‘visual research methods’ are better methods.
   b. The comparative discussion should not be between quantitative vs qualitative methods but participatory or visual methodologies.
