# Supplementary material for: Female adolescents living with HIV telling their story through ‘my story’ book in Malawi: A visual methodology innovation
Source: PLoS One. 2021 Oct 15;16(10):e0257126. doi: 10.1371/journal.pone.0257126 (PMC8519441; doi:10.1371/journal.pone.0257126)
Supplement: Attachment — Submitted filename: RESPONSES TO REVIEWER 2 s COMMENTS 190721.docx [file pone.0257126.s007.docx]

**RESPONSES ON COMMENTS AND OBSERVATIONS MADE ON THE MANUSCRIPT**

**BY REVIEWER 2**

| **SECTION** | **COMMENTS/OBSERVATIONS** | **RESPONSE** | **PAGE NUMBER** |
| --- | --- | --- | --- |
|  | - There are a few paragraphs where the authors repeat their ideas. a. Pages 9 and 19 when the authors refer to ‘safe places’. b. Pages 9 and 10 as they talk about ‘keeping the thematic log.’ | All repetitions have been rephrased/ removed throughout the document |  |
| Introduction | - The paragraphs in the introduction need to be strengthened. I appreciate the arguments from Kellett, Chambers and the appropriateness of using the ‘my story book’. a. There is a need to know more about the concept of ‘my story book’ or the ideas of ‘memory book’. b. How did the ‘book’ work in the past? How they have been used global, sub-Saharan Africa and Malawi are still missing. c. ‘How?; When? and where? These concepts have been used, will strengthen the gap that Teachman and colleagues and Wills and colleagues presented. d. As it is, the paragraphs from pages 4-7. i. The concept of my story books… ii. The main themes of my story books… iii. The discussion held with the researcher. The above paragraphs leave the reader hanging and need real-life supporting evidence from the region or elsewhere. In addition, they continue to read like a paragraph that is part of the methodology section rather than the introduction. We need a solid and convincing introduction section. | This information is included and the section is strengthened  These paragraphs have been moved to methodology section as suggested by the first reviewer  Convincing introduction included | 3-5  8-9 |
| Methodology | - The methodology is still too long and it shares some results too. a. On page 7, the authors refer to 14 adolescents who completed the story book. b. The researchers/author’s positionality should be reflected as part of data collection rather than recruitment and sampling. | The results have been moved  Responding to comments/suggestions/ requests from two reviewers makes this section too long - paragraphs are moved from other sections to here.  Fourth paragraph under recruitment and sampling has been moved to results.  Last paragraph has been rephrased and considered as justification for the age of 15-19 for the adolescents | 12  12  6 |
| Discussion | - . Please include tangible evidence from the region (sub-Saharan Africa) on how using memory books/my story book or ‘visual research methods’ are better methods. b. The comparative discussion should not be between quantitative vs qualitative methods but participatory or visual methodologies. | There paucity in literature on ‘My story’ book because it is a novel data collection method.  This is not a discussion but a limitation of the full study so it has been removed | 18 |
